# Supplementary material for: Causal Relationship between Aging and Anorexia Nervosa: A White-Matter-Microstructure-Mediated Mendelian Randomization Analysis
Source: Biomedicines. 2024 Aug 16;12(8):1874. doi: 10.3390/biomedicines12081874 (PMC11351342; doi:10.3390/biomedicines12081874)
Supplement: Supplementary file 1 [file biomedicines-12-01874-s001.zip › Supplementary Figure/Figure S8/Leaveoneout plot of MR analysis for TL to ubm-b-2064.pdf]

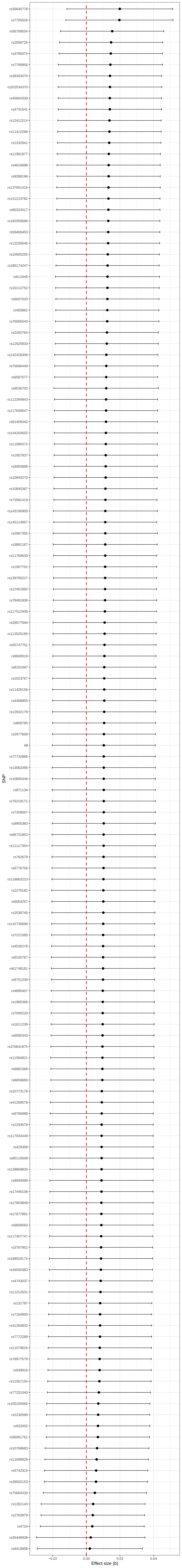

rs35640778
rs7705526
rs56799554
rs2056726
rs3785074
rs7790856
rs28363070
rs202034370
rs45604339
rs4731541
rs12412214
rs11412296
rs1332941
rs11991877
rs4616688
rs9398196
rs137901416
rs141214782
rs80324517
rs182059586
rs59409453
rs13230646
rs10905255
rs185174247
rs611646
rs10112752
rs6007020
rs450962
rs76065543
rs2282764
rs12925933
rs142426306
rs76666449
rs6587577
rs6536702
rs112394943
rs117630647
rs61405042
rs144204502
rs11085072
rs1957937
rs3093888
rs10840270
rs10845387
rs73581419
rs143190905
rs145114957
rs2967355
rs3891167
rs11769630
rs1907702
rs139795227
rs12451892
rs78491606
rs117512405
rs28577594
rs113525195
rs55747751
rs9600019
rs8102497
rs1023767
rs11426156
rs4498805
rs12932179
rs869785
rs2977608
All
rs77732866
rs13062095
rs10805346
rs871134
rs76219171
rs7209057
rs9955360
rs66731853
rs11117354
rs762679
rs6776756
rs116863223
rs2276182
rs60054257
rs2538745
rs142730696
rs7221585
rs4530278
rs8105767
rs61748181
rs6751209
rs4695407
rs1985369
rs7099229
rs1611236
rs6590343
rs376641875
rs11584821
rs6881568
rs6659669
rs10773176
rs41269079
rs6790988
rs2293579
rs117034449
rs429358
rs80116508
rs139669835
rs9940099
rs17445108
rs17803849
rs17677991
rs6669563
rs117407747
rs3767952
rs189918174
rs34550383
rs4743037
rs11212631
rs131797
rs7164950
rs41304832
rs7772289
rs11579626
rs79977579
rs939916
rs11557154
rs77231040
rs150150565
rs2230590
rs932002
rs56061761
rs10768683
rs11699829
rs5742915
rs28502153
rs75664430
rs1291143
rs2763979
rs4724
rs35446936
rs9419958
SNP
-0.02
0.00
0.02
0.04
Effect size (b)
